# Severe Hyponatremia (96 mmol/L) Secondary to Primary Polydipsia and Pneumonia

**DOI:** 10.7759/cureus.62915

**Published:** 2024-06-22

**Authors:** Nikeeta Mandhan, Michael Schaible, Howard Yu, Sahil Chaddha, Huma Ahmed, Robert Foronjy

**Affiliations:** 1 Internal Medicine, State University of New York Downstate Medical Center, Brooklyn, USA; 2 Pulmonary and Critical Care, State University of New York Downstate Medical Center, Brooklyn, USA

**Keywords:** altered mental state, aspiration pneumonia, intractable hiccups, primary polydipsia, acute hyponatremia

## Abstract

A 63-year-old man who presented to the hospital with altered mental status and decreased responsiveness was found to have severe symptomatic hyponatremia with a sodium level of 96 mmol/L and pneumonia. The patient was admitted to the medical intensive care unit for septic shock and acute severe hyponatremia. He was intubated for airway protection, and treated with 3% hypertonic saline bolus and antibiotics. After four days, sodium levels were corrected to 128 mmol/L, and the patient was extubated and downgraded to the medical floor. This case demonstrates one of the lowest recorded sodium lab values ever and the patient was successfully treated and discharged home with appropriate outpatient appointments.

## Introduction

Hyponatremia is the most common electrolyte abnormality affecting 35% of hospitalized patients and is defined as a serum sodium level less than 135 mmol/L [1]. The symptoms are based on the severity of hyponatremia and range from mild nausea and confusion to coma and seizure [2]. The prevalence of hyponatremia in the intensive care unit is approximately 15% and is an independent predictor of mortality [3]. There are a few case reports of symptomatic hyponatremia of sodium <100 mmol/L in adults: a case of hyponatremia secondary to valproic acid overdose with a serum sodium level of 99 mmol/L [4], a case of hyponatremia secondary to excessive thiazide diuretic use with a serum sodium level of 99 mmol/L [5], and a case of hyponatremia secondary to seizure disorder and recent alcohol consumption with a serum sodium level of 95 mmol/L measured at an outside hospital [6]. We report a case with a serum sodium level of 96 mmol/L secondary to primary polydipsia and pneumonia.

## Case presentation

A 63-year-old man with a history of atrial fibrillation, gastroesophageal reflux disease, and insulin-independent diabetes mellitus was brought to the emergency department for altered mental status and decreased responsiveness 11 hours prior to admission without any preceding symptoms. The patient has a 30-year history of intractable hiccups, which have been refractory to medical and surgical management including phrenic nerve blocks. The patient's hiccups worsened 10 days prior to admission, leading him to self-vomit and consume 10-12 L of diet ginger ale daily to relieve symptoms. The family denies a history of headache, fever, head trauma, slurred speech, unilateral weakness, a witnessed aspiration, or diarrhea. 

**Figure 1:**
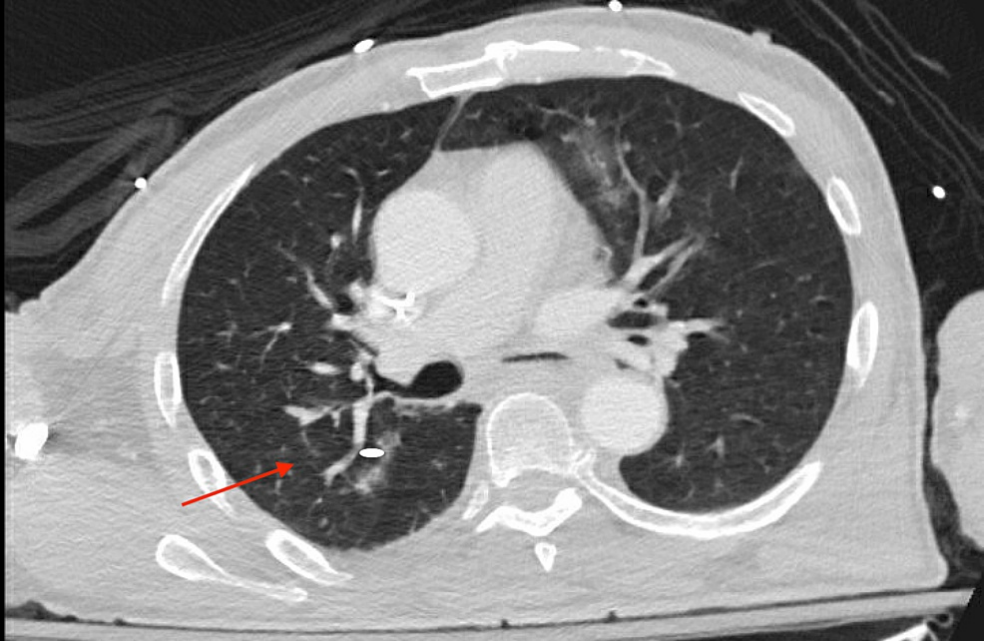
CT chest with contrast depicting multifocal alveolar disease, predominantly ground-glass opacification most severe in the left upper lobe. Mild tree-in-bud centrilobular nodularity identified in the right upper lobe (arrow)

On admission, the patient was afebrile with a blood pressure of 181/106 mmHg, heart rate of 76 beats per minute, and saturating 98% on room air. The patient was euvolemic, had a Glasgow Coma Scale of 4, was not in respiratory distress, demonstrated partial right-sided gaze palsy with no doll’s phenomenon, and was hiccupping. Labs were suggestive of hyponatremic hypokalemic hypochloremic metabolic alkalosis: sodium (Na) 96, potassium (K) 2.5, and chloride (Cl) 59 (Table 1). Urine toxicology was negative. Urine studies were consistent with attempted sodium retention: serum osmolarity was 212 (275-295 mOsm/kg), urine sodium was 22 (30-90 mmol/L), and urine osmolarity was 272 (500-800 mOsm/kg). Cortisol was 8.8 (4.0-19.0 mcg/dL) and thyroid-stimulating hormone was 0.45 (0.38-4.70 ulU/mL) and they were unremarkable. The patient had leukocytosis with a white blood cell level of 15 (3.50-10.80k/uL) (Table 2). A CT chest reported left upper lobe ground glass opacity (Figure 1), which was supported by positive urine streptococcus antigen and was consistent with a diagnosis of pneumonia. The patient was admitted to the medical intensive care unit for acute hyponatremia and pneumonia, was started on a 3% hypertonic saline bolus of 100 mL, and a central line was placed.

**Table 1:** Electrolytes throughout hospitalization

Electrolyte	Reference Range (mmol/L)	Day of Hospitalization									
		1	1	1	1	1	2	3	3	3	4
Sodium	136-145	96	99	110	105	106	108	110	115	123	128
Potassium	3.5-5.1	2.5	2.7	2.1	3.1	2.8	3.4	3.2	3.5	4.1	4.3
Chloride	98-107	59	61	75	72	70	77	76	81	90	89

**Table 2:** Day 1 hyponatremia labs

Unit of Interest	Reference Range	Day 1 Value
Serum osmolarity (mOsm/kg)	275-295	212
Urine sodium (mmol/L)	30-90	22
Urine osmolarity (mOsm/kg)	500-800	272
Cortisol (mcg/dL)	4.0-19.0	8.8
Thyroid-stimulating hormone (ulU/mL)	0.38-4.70	0.45
White blood cell count (k/uL)	3.50-10.80	15

**Figure 2:**
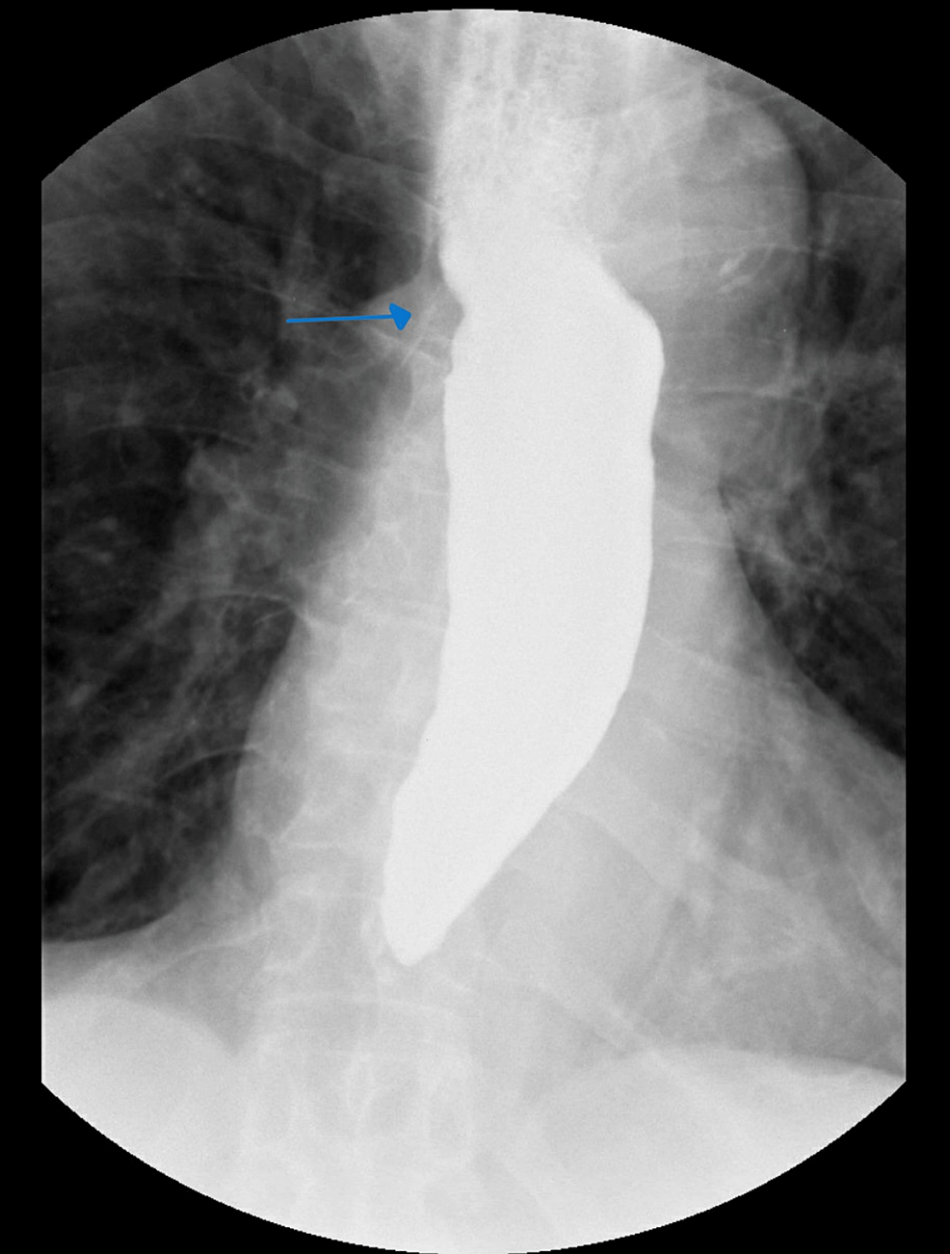
Barium swallow demonstrating esophageal dysmotility with marked delayed esophageal emptying and a small hiatal hernia (arrow)

Due to altered mental status and concern of nonconvulsive status epilepticus, 3 mg lorazepam was given along with a levetiracetam loading dose. Although he was initially protecting his airway, after the first bolus of 3% saline, there was no change in his mental status and he had an episode of emesis. He was then intubated for airway protection. A five-day course of vancomycin and piperacillin and tazobactam was started for pneumonia. The goal sodium correction was 6-8 mmol/L in the first 24 hours. Sodium was overcorrected to 110 mmol/L, and desmopressin and 5% dextrose and half concentration of normal saline were initiated. The patient's sodium level was corrected with fluid restriction and electrolyte supplementation. Within four days, sodium levels increased to 128 mmol/L, and the patient was successfully extubated on day 5 (Table 1). The speech language pathology bedside swallow evaluation found the patient to have oral phase dysfunction but the patient passed the trial of liquids and the diet was restarted. Barium swallow was significant for dysmotility with marked delayed esophageal emptying and a small hiatal hernia was noted (Figure 2). There were no ulcers, strictures, or masses seen. The patient was started on a proton pump inhibitor and was discharged home with outpatient gastroenterology, nephrology, and primary care follow-up. 

The patient was subsequently rehospitalized for similar complaints six months later and was treated for symptomatic hyponatremia when the sodium level was 116 mmol/L.

## Discussion

This patient presented with altered mental status, right gaze deviation, and intractable hiccups. The patient's hiccups were refractory to medical and surgical management, which caused him to drink about 10 L of excess liquid. The history and initial labs were suggestive of primary polydipsia. Subsequent imaging, CT chest, and urine culture confirmed a secondary diagnosis of strep pneumonia. Polydipsia along with pneumonia both contributed to severe hyponatremia.

The patient relieved his hiccups with excessive liquid consumption and self-induced vomiting. A hiccup is an involuntary myoclonic contraction of the diaphragm and inspiratory muscles. Contraction of intercostal muscles is followed by closing of the glottis leading to the characteristic "hic" sound of hiccups. The nerve signal is transduced through the vagus, phrenic, intercostal, and recurrent laryngeal nerves to close the glottis [7]. Hiccups are more common in the central nervous system and gastrointestinal pathologies.

Hiccups can be acute, persistent, or intractable. An acute hiccup lasts less than 48 hours. Persistent hiccups last between two days and one month. Intractable hiccups last more than one month. The Guinness Book of Records documents the longest period of continuous hiccupping as 68 years by a man who fell and injured a small blood vessel in the brain which inhibited his hiccup response [8]. Although usually a benign and self-limited annoyance, persistent hiccups may be a sign of serious underlying illness [9]. Induced vomiting is one of the physical therapies known to mitigate hiccups. The patient had both salt loss, through vomiting, and polydipsia which led to symptomatic severe hyponatremia.

Altered mental status, seizures, and comatose state are common symptoms when the serum sodium falls abruptly [10]. Seizures are uncommon in chronic hyponatremia, even at extremely low serum sodium concentrations, because the brain can adjust osmotically active solutes over time to correct for low salt concentrations in the extracellular space [11]. Response to antiepileptic medications can be poor unless the serum sodium concentration is increased [12]. 

Symptomatic hyponatremia is treated with small, fixed boluses of hypertonic saline as soon as possible, according to recent American and European guidelines. It is currently advised to correct hyponatremia slowly to prevent osmotic demyelination syndrome (ODS) [13]. In ODS, the rapid change in salt concentration leads to dehydration and demyelination of the nervous sheath leading to permanent brain damage [14]. Patients at a high risk of ODS should receive slower serum sodium correction. A 2021 study from Geisinger determined that patients with liver disease, beer potomania, hypokalemia, and sodium <105 mol/L are at increased risk for ODS. Correction of sodium should not exceed 8 mol/L in the first 24 hours. The treatment of choice for hyponatremia is boluses of hypertonic saline instead of a continuous infusion [15]. 

SALSA randomized controlled trial in 2021 compared the risk of overcorrection with the use of rapid intermittent bolus (RIB) and slow continuous infusion (SCI) with hypertonic saline in patients with symptomatic severe hyponatremia. Compared to the SCI group, the RIB group received a larger dose of hypertonic saline (3%), which was provided within 1 and 6 hours. The RIB group had a higher percentage of patients who achieved a target correction rate in less than an hour, with lower chances of overcorrection. RIB therapy was more effective for 1 hour. Contrary to RIB, SCI carries a 10%-16% risk of unintentional overcorrection [16].

## Conclusions

The case described here documented one of the most severe cases of acute hyponatremia with a serum sodium lab of 96 mmol/L. This presentation arose from a combination of primary polydipsia, self-induced vomiting, and underlying pneumonia. The patient was successfully treated with hypertonic saline boluses, fluid restriction, and antibiotics and ultimately discharged home with a sodium level of 135 mmol/L. This case highlights an extreme example of how critically ill patients require multidisciplinary approaches to address complex medical diseases. 
